# Traffic-Related Air Pollution and Asthma Onset in Children: A Prospective Cohort Study with Individual Exposure Measurement

**DOI:** 10.1289/ehp.10968

**Published:** 2008-06-18

**Authors:** Michael Jerrett, Ketan Shankardass, Kiros Berhane, W. James Gauderman, Nino Künzli, Edward Avol, Frank Gilliland, Fred Lurmann, Jassy N. Molitor, John T. Molitor, Duncan C. Thomas, John Peters, Rob McConnell

**Affiliations:** 1 School of Public Health, Division of Environmental Health Science, University of California, Berkeley, California, USA; 2 Department of Preventive Medicine, Keck School of Medicine, University of Southern California, Los Angeles, California, USA; 3 Center for Research in Environmental Epidemiology (CREAL), Institut Municipal d’Investigació Mèdica (IMIM), Barcelona, Spain; 4 Sonoma Technology Inc., Petaluma, California, USA; 5 Department of Epidemiology and Public Health, School of Medicine, Imperial College, London, UK

**Keywords:** air pollution, asthma onset, children, nitrogen dioxide

## Abstract

**Background:**

The question of whether air pollution contributes to asthma onset remains unresolved.

**Objectives:**

In this study, we assessed the association between asthma onset in children and traffic-related air pollution.

**Methods:**

We selected a sample of 217 children from participants in the Southern California Children’s Health Study, a prospective cohort designed to investigate associations between air pollution and respiratory health in children 10–18 years of age. Individual covariates and new asthma incidence (30 cases) were reported annually through questionnaires during 8 years of follow-up. Children had nitrogen dioxide monitors placed outside their home for 2 weeks in the summer and 2 weeks in the fall–winter season as a marker of traffic-related air pollution. We used multilevel Cox models to test the associations between asthma and air pollution.

**Results:**

In models controlling for confounders, incident asthma was positively associated with traffic pollution, with a hazard ratio (HR) of 1.29 [95% confidence interval (CI), 1.07–1.56] across the average within-community interquartile range of 6.2 ppb in annual residential NO_2_. Using the total interquartile range for all measurements of 28.9 ppb increased the HR to 3.25 (95% CI, 1.35–7.85).

**Conclusions:**

In this cohort, markers of traffic-related air pollution were associated with the onset of asthma. The risks observed suggest that air pollution exposure contributes to new-onset asthma.

Toxicologic and epidemiologic research suggests that air pollution exacerbates asthma symptoms (Delfino 2002; Eder et al. 2006), but few prospective studies have addressed the question of whether this pervasive exposure contributes to disease onset in children. Recent research has focused on the contribution of traffic-related air pollution, partly due to the toxicologic effects of the pollution mixture from mobile sources (Nel 2005). Studies based on prevalent asthma have been inconsistent, with some reporting positive associations between traffic-related exposure and asthma or associated symptoms (Brunekreef et al. 1997; Ciccone et al. 1998; Duhme et al. 1996; Edwards et al. 1994; Gauderman et al. 2005; McConnell et al. 2006; Migliaretti and Cavallo 2004; Nitta et al. 1993; Oosterlee et al. 1996; van Vliet et al. 1997; Weiland et al. 1994; Wjst et al. 1993) and others reporting no significant associations (Lewis et al. 2005; Livingstone et al. 1996; Wilkinson et al. 1999).

A few prospective studies have assessed the relation between traffic pollution and asthma onset (Brunekreef and Sunyer 2003; Eder et al. 2006; Gold and Wright 2005). A case–control study from France that selected subjects by medical clinic found that early-life exposure to traffic pollutants may contribute to asthma incidence (Zmirou et al. 2004). In a prospective birth cohort study from the Netherlands, traffic-related pollutants were associated with incident wheezing and doctor-diagnosed asthma in young children up to 4 years of age (Brauer et al. 2002, 2007). An earlier study from Japan showed positive associations between community nitrogen dioxide levels measured at a central monitor and asthma incidence in school children (Shima et al. 2002). None of these studies used individually based measures of exposure, and modeled exposures may have large errors that attenuate effects (Molitor et al. 2006). In this context, we investigated the relationship between childhood asthma onset and measured markers of traffic-related air pollution in Southern California.

## Materials and Methods

### Sample selection

We selected a sample of 217 children from 917 eligible subjects living in 11 communities in the Southern California Children’s Health Study (CHS), a prospective cohort study of air pollution and respiratory health. Subjects were selected randomly from the larger cohort based on different levels of traffic-related exposure. Specifically, we divided subjects into two strata (above and below the median traffic exposures within each community) and then selected an equal number randomly from each of the strata. Although our study is similar to an earlier cross-sectional study from this population that focused on the effect of freeway-related traffic in 10 CHS communities (Gauderman et al. 2005), in this incident asthma analysis we excluded children with a lifetime history of asthma at study entry, and we included an 11th community, Lompoc, which had local traffic influence but no major freeways. A 12th community, Lake Arrowhead, had little local traffic variability and was not included in either analysis.

Case ascertainment was as described previously (Gauderman et al. 2005; McConnell et al. 2002a). We excluded children with prevalent asthma from this analysis if a parent answered yes to the question “Has a doctor ever diagnosed this child as having asthma?” on a baseline survey sent home with children at the time of study enrollment (at 10 years of age) in 1993 or 1996. We also excluded children who answered yes to the question “Has a doctor ever said you had asthma?” on a questionnaire administered during the first year of the study by a trained interviewer. We then classified as incident cases children who answered yes to this question on any annual interview during up to 8 years of follow-up until high school graduation. Information on demographic and other known risk factors for asthma development, such as pets, molds in the home, and environmental tobacco smoke, was obtained at baseline and updated annually. We obtained informed consent from parents, and the study was approved by the University of Southern California Institutional Review Board.

### Exposure assignment

As described previously (Gauderman et al. 2005), children had NO_2_ monitors (Palmes tubes; Gradko, Winchester, UK) placed in the front or back yard of their home in the year 2000 for 2 weeks in the summer (mid-August) and 2 weeks in the fall–winter season (mid-November). We deployed all monitors within 4 days to minimize temporal variation of the exposure estimates by change of season or meteorologic events. Within each community, monitors were deployed within a 4-hr period. We obtained valid readings for 209 subjects for the ambient household level of NO_2_ measurements during fall–winter months and 204 for summer months. We assessed exposures by season and as an average of the two measurement periods.

### Contextual confounding variables

We also derived a series of potential “contextual” confounders at the community level. Contextual characteristics of the community, particularly socioeconomic position (SEP), have been associated with respiratory health effects (Haan et al. 1987; Jin et al. 1994; Juhn et al. 2005; Wissow et al. 1988). For this study, we tested the SEP measures of median household income, proportion of respondents with low education (i.e., no high school diploma), percent of males unemployed (as a marker for full-time income instability), and percent living in poverty as confounders. We used male unemployment (as opposed to general or female unemployment) because the relationship between employment and health is stronger for males than for females (Jin et al. 1994; Sorensen and Verbrugge 1987). Sensitivity analyses were also conducted with total unemployment. Definitions of unemployment and poverty followed those developed by the U.S. Census Bureau (1992). We used these variables to determine whether variation in asthma could be explained by the social conditions in the neighborhood because such contextual effects have been suggested as another risk factor for asthma incidence (Gold and Wright 2005).

Characteristics of SEP were aggregated from census blocks where study subjects lived. We used data from the U.S. 1990 census to estimate community conditions at the time of study enrollment, which began in 1993. Because we recruited subjects through schools, residential locations clustered together. To compensate for clustering of study subjects, we weighted these community-level data by the proportion of the census blocks included in a community-specific bounding rectangle that contained 95% of local study subjects (Tatalovich et al. 2006). This spatial bounding captures the social characteristics around the residence of the children but excludes a few spatial outliers.

### Meteorologic variables

Meteorologic conditions, especially temperature and humidity, may also be potential risk factors for asthma (Guo et al. 1999; Weiland et al. 2004). Based on data collected daily in 1995 (close to the midpoint of baseline enrollment) from local meteorologic stations in each community, we derived 10 markers of humidity and temperature, including minimum and average temperature in winter and summer months, maximum and average humidity in winter and summer months, and annual average temperature and humidity. We tested these as possible confounders of the asthma–air pollution relationship.

### Statistical model

We used random-effects Cox proportional hazards models to assess the risk of asthma onset in relation to pollution exposures, while stratifying for age in years and sex in the baseline hazard. Community-level random effects allowed for clustering and assessment of residual community variation in time to asthma onset (Jerrett et al. 2005; Ma et al. 2003). The model took the following form:


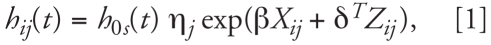


where *h_ij_*(*t)* is the hazard function for the *i*th subject in *j*th community, *h*_0_*_s_*(*t)* is the baseline hazard function for stratum *s* (i.e., age and sex), η*_j_* represents the positive random effects for community *j* with expectation 1 and variance σ^2^, *X_ij_* is the air pollution exposure (i.e., NO_2_) for individual *i* in community *j*, and *Z_ij_* represents covariates (e.g., health insurance) for individual *i* in community *j*.

We evaluated pollution exposures with other covariates such as relative humidity and medical insurance, which we treated as confounders if they had an association with asthma incidence and changed the pollution coefficient by ≥ 5% for the annual NO_2_. We report results across the average within-community interquartile range of annual average NO_2_ exposure.

To determine whether exposures could be assessed at the individual level across the entire range, we tested for significant differences in effects from community aggregated means from the individually assigned exposure deviated from those community means. The model that allows for separate between-community means and the within-community deviations had the following form:


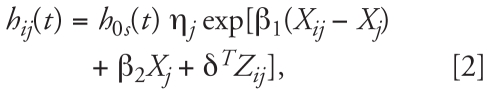


where *X_ij_* is the individually assigned exposure (i.e., NO_2_) and *X_j_* is the community average of the individual measurements. We then assessed the equivalence of these two pollution effects by testing the hypothesis H_0_: β_1_ = β_2_. If the two exposure β values were not statistically different based on the log-likelihood ratio test, we concluded that there was no need to distinguish between the within-community and between-community effects of pollution.

We examined this alternative model to determine whether the individual exposures specified in Equation 1 were appropriate or whether we needed a model specifying different within- and between-community effects for correct interpretation of results.

## Results

Table 1 lists the descriptive statistics and bivariate associations with asthma for the sample and all the variables used in the analysis. Hispanic Americans and those of white race were well represented in this sample, but there were few Asian Americans, African Americans, or others. Males represented just about 40% (86) of this sample. Many of the children had potential risk factors for asthma in the home, with 95% (183) having carpet in the child’s bedroom, 83% (181) having a pet, and 79% (168) having a gas stove. About 17% (35) of the children had a parental history of asthma. The median age at baseline was 9.6 years. Except for a higher proportion of females, there were no significant differences between the frequency of these variables among the subjects of this study and the remainder of eligible subjects from which we drew the study sample. The study sample and other eligible subjects also had similar traffic exposures (results not shown).

The bivariate associations of individual and household variables as predictors of asthma were largely not statistically significant (Table 1). Associations with body mass index (BMI) and other covariates were generally consistent with associations observed in previous analyses of the entire cohort from which we drew this study sample (Gilliland et al. 2003; McConnell et al. 2002a). In this sample, Hispanics were more likely to report incident asthma during the follow-up than were whites. Because of small counts, we could not derive stable estimates for African Americans. Less than a third of the homes had mildew or a smoker, which may have led to unstable estimates.

Table 2 shows the contextual confounders tested in the modeling process. In general, lower community SEP was associated with higher risk for asthma onset. Higher winter temperature was associated with higher risk for asthma. The effects of temperature were less consistent than those for humidity, which was a risk factor in all seasons. Higher relative humidity was generally associated with higher risk for incident asthma.

**Figure 1:**
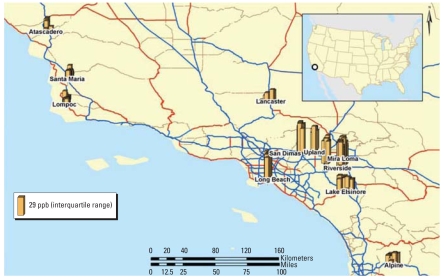
Measured NO_2_ at subjects’ homes in the 11 study communities: summer/winter average.

In Figure 1, the bars illustrate individual residential NO_2_ levels within each study community. Variation exists within communities, and a broader regional pattern is also present. Communities in the Los Angeles metropolitan area have generally higher levels than do communities to the north and south, showing a spatial pattern consistent with earlier measurements based on central monitors (Gauderman et al. 2004). We examined the incidence rate by tertile of exposure. Dividing the sample into tertiles of annual NO_2_, the crude asthma incidence rates, from lowest to highest tertile of exposure, were 14.2, 19.1, and 20.7 per 1,000 person-years, respectively [with wide confidence intervals (CIs)].

Table 3 presents the descriptive statistics for the exposure variables within each community. There was a wide range of average exposure across communities but also substantial variation within communities (mean interquartile range of 6.2 ppb). Communities in the Los Angeles metropolitan area (Lake Elsinore, Long Beach, Mira Loma, Riverside, San Dimas, and Upland) generally had higher NO_2_.

We tested each of the individual variables shown in Tables 1 and 2 with an association to asthma to determine whether confounding reduced the pollution coefficient by ≥ 5%. Table 4 contains the results of the final models, in which we retained Hispanic ethnicity, medical insurance, enrollment group, and relative humidity as confounding adjustment variables. All pollution metrics were positively associated with incident asthma. For average residential NO_2_ the hazard ratio (HR) was 1.29 (95% CI, 1.07–1.56) across the average within-community interquartile range of 6.2 ppb. The fall–winter HR of 1.29 (95% CI, 1.11–1.49) was nearly the same as the annual average, whereas the summer HR of 1.27 (95% CI, 1.03–1.59) was slightly smaller. The sample size was 196 with 26 cases (for the annual NO_2_), so it is possible that influential outliers might have affected the results. However, there were no cases with exceptionally high exposure to NO_2_ and no noncases with notably low exposure to NO_2_.

The σ^2^ values are the random-effects variance estimates, which represent the residual variation in asthma incidence attributable to the community cluster after we included all other fixed predictors in the model (Table 4). Addition of the individual covariates in model 1 reduced the σ^2^ only slightly from a null model containing only baseline age and sex strata (results not shown). Models 2 and 3 show the large decline in σ^2^ when we included community relative humidity and pollution, respectively, indicating that both variables account for a large proportion of the residual between-community variation in asthma incidence.

We assessed exposures both as the deviation of the individual specific exposure metric from the corresponding community mean and as the community average (i.e., average of household NO_2_ per community). We used these variables to compare within- and between-community pollution effects. Table 5 lists results of the tests shown in Equation 2 for difference in the between-community exposures and deviation within community exposures. The between-community summer NO_2_ point estimate was larger than the within-community estimate, but these estimates had large CI values and were not significantly different based on the likelihood ratio test. For yearly average and winter NO_2_, the within- and between-community effects were similar.

Interactions between humidity and air pollution were tested, but found not to be significant, although pollution did appear to exert larger effects in more humid areas (*t*-values were between 0.9 and 1.3 depending on the humidity interaction term with various measures of pollution).

We conducted further sensitivity analyses to assess whether excluding early wheeze at baseline affected the results (Table 6). Excluding baseline wheeze had a small impact on the asthma effect of air pollution. We implemented another sensitivity test by removing data for all children with a history of early chest illness, including croup, bronchitis, bronchiolitis, or pneumonia (Table 6). The HR values were mildly attenuated but still elevated and similar to the HR values without these exclusions.

## Discussion

In this prospective cohort of children from 11 Southern California communities, we found significant associations between incident asthma and measured outdoor residential exposure to NO_2_. A strength of the study was the individual assignment of exposure, based on measurements of NO_2_ outdoors at the homes of the children. Previous research on incident asthma and air pollution has largely relied on modeled exposure (Brauer et al. 2002; Zmirou et al. 2004) or on exposure measured at central site locations (McConnell et al. 2002a; McDonnell et al. 1999; Shima et al. 2002). To our knowledge, this is the first study to employ individually measured pollutant exposures with incident disease assessment. Although the results must be interpreted in light of the limited sample size and small number of cases, they highlight a need for further examination of relationships between incident disease and individually measured exposures.

We tested numerous individual and contextual confounding variables. Some of the variables that had an association with asthma were not confounders because they exerted little or no effect on the pollution coefficient. Parsimonious models controlling for age and sex in the baseline hazard indicated that Hispanic ethnicity, medical insurance, and relative humidity were confounders in models with NO_2_. The air pollution exposures generally accounted for larger portions of the residual variance in the community random effects (i.e., σ^2^) than did the individual-level variables included as confounders. The results for residential NO_2_ were sensitive to inclusion of the relative humidity variable. This variable met our criteria for confounding and increased the pollution effect (Table 4). Because adjustment for humidity elevated the risks associated with air pollution exposure, we further investigated an interaction between pollution exposures and humidity. Although the interaction was not significant, we did observe generally higher risks for pollution in more humid communities.

In the absence of interaction, the influence of humidity may represent a case of variance suppression (Tabachnick and Fidell 1985), whereby confounding variables may increase the effect size of the primary risk variable in the model. In this case, relative humidity and the pollutants share some variance, and this shared component also partially overlaps with the asthma outcome. The part of the variance shared by relative humidity and pollution is the component of variance with asthma that has a relatively less precise association with pollution. With the inclusion of relative humidity, this less precise component of the variance overlap between pollution and asthma is removed, and the remaining relationship becomes more pronounced.

We further investigated the possibility of positive confounding by examining the correlation between humidity and NO_2_ and the impact of the humidity risk with NO_2_ excluded from the model. There was a moderately high, significant negative correlation between NO_2_ and relative humidity. Evaluated at the household level for the annual estimates, the Pearson correlation coefficient was −0.57, and at the community average level of NO_2_ it was −0.61. Except for Long Beach, most of the coastal communities had lower air pollution and higher humidity. Because humidity was positively associated with asthma but negatively associated with NO_2_, we would expect humidity to be a negative confounder (i.e., excluding humidity from the model should result in a negative bias on the NO_2_ estimate). In this case, we did observe a negative bias when humidity was not included in the model. We would also expect a negative bias in the humidity effect when we exclude NO_2_ from the model. Table 7 shows the humidity risks with and without NO_2_ in the model. Without NO_2_, the risks were attenuated in all models, and the negative bias was strongest in the summer season. Thus, the impact of humidity was negatively biased when we excluded NO_2_ from the model, which is consistent with the positive confounding effect observed in the NO_2_ models. These results imply that simultaneous consideration of meteorology and air pollution may be important in models assessing the effects of chronic air pollution exposure on respiratory disease.

Interpreting the household NO_2_ findings is complicated because it represents a mixture of local and regionally transported pollutants in Southern California. In an earlier analysis, Gauderman et al. (2005) reported moderate to high correlations between the measured residential NO_2_ and various measures of traffic proximity or modeled concentrations. Although some toxicologic evidence supports the potential for NO_2_ as a direct causative agent for respiratory health effects at high concentrations (Kelly and Sandstrom 2004), the more likely interpretation is that NO_2_ was a proxy for other components of the local air pollution mixture with high toxic potential. Because NO_2_ pollutants may be transported from neighboring areas, some of the effects observed could represent local and regional contributions. If NO_2_ represents other toxic traffic-related pollutants, the transported component would have different toxicologic properties than purely local contributions.

Rather than the specific causal agent, NO_2_ may represent a mixture of local traffic-related pollution and regional pollutants, which in Southern California include transported primary traffic-related pollutants and regional products of photochemistry. The design of the study allowed us to examine the independent effects of each of these contributions to the pollutant mixture by modeling the effects of the within- and between-community variation in NO_2_, as shown in Table 5. The within-community effects indicative of long-term local traffic sources were similar in magnitude to effects of community average NO_2_ across communities, suggesting that both regional and local pollution contributed to the associations with asthma. However, the range of variation of NO_2_ within communities was smaller than between communities, and the HR values were therefore smaller when constrained to the range within communities. For example, using the average interquartile range across all measurements of 28.9 ppb for annual NO_2_ increased the HR to 3.25. For the average within-community range of 16.4 ppb, the HR was 1.95.

Compared with earlier studies on prevalent asthma, with a similar population and the same exposure measurements for NO_2_, the odds ratio per interquartile range increment of 5.7 ppb was 1.83 for prevalent asthma (Gauderman et al. 2005). Thus, the incident effects were smaller than those observed for prevalent asthma over a similar exposure contrast. The overall effects we observed here nonetheless support results from our earlier prevalence study. In both studies, we observed effects on asthma from NO_2_.

Notable strengths of this study are the prospective design, individually measured pollutant exposures, incident disease assessment, and the large effects. Limitations include an inability to identify the specific constituents of pollution that were responsible for the observed health effects, the relatively small sample size, and questionnaire case ascertainment. Although a potential limitation, self-reported physician diagnosis is widely used to define asthma in epidemiologic studies (Burr 1992), is reproducible (Ehrlich et al. 1995; Peat et al. 1992), and reflects what physicians actually reported to patients (Burney et al. 1989; Greer et al. 1993). We conducted sensitivity analyses with alternative case definitions, excluding children with baseline wheeze and early childhood chest illness. The summer estimates were attenuated mildly (HR values range from 1.21 to 1.26), and after excluding baseline wheeze became insignificant (Table 6). Many of the communities had lower NO_2_ levels in the summer (Table 3), and the effects during the summer season were smaller than in the other seasons. In contrast, the HR values for NO_2_ were only slightly smaller for the fall–winter and annual estimates and remained significant (HR values range from 1.2 to 1.9). The general conclusions, therefore, are similar even after excluding children with wheeze at baseline or with a history of early childhood illness.

The overall asthma incidence rate per thousand person-years of 18.8 cases resulting from parent- or child-reported physician diagnosis was high but is similar to rates reported previously in the CHS cohort (McConnell et al. 2002b) and in other studies of asthma or wheeze in school children (Lombardi et al. 1997; Ronmark et al. 2001). Lower rates have been found in other studies (Broder et al. 1974; Dodge and Burrows 1980; Yunginger et al. 1992). Comparison across studies, however, is complicated by varying ascertainment methods and temporal trends in incidence rates. The rate in this sample from the larger CHS cohort was similar to those in comparable studies.

Further field studies measuring plausibly causal ultrafine particles, metals, and poly-cyclic aromatic hydrocarbons simultaneously (Nel 2005) might identify the constituents responsible for the observed association with NO_2_. Although the specific pollutant or related mixture of constituents responsible for the effects remains the focus of future research, the large risks observed suggest that traffic-related air pollution exposure contributes to new-onset asthma.

## Figures and Tables

**Table 1:** Subject characteristics and bivariate associations with asthma onset.

Risk factor	No. (%)^a^	Hazard ratio (95% CI)^b^
Subject characteristics		
Race/ethnicity		
Hispanic ethnicity	66 (32)	2.15 (1.00–4.58)
African-American	3 (1.5)	NA^c^
Asian	14 (6.8)	1.05 (0.20–5.61)
White non-Hispanic^d^	120 (59)	1.00
Mixed/other race	14 (6.5)	0.42 (0.04–4.57)
BMI^e^		
Below 10th percentile	27 (12)	0.54 (0.14–2.08)
10^th^–90th percentile^d^	177 (82)	1.00
90^th^ percentile	13 (6.0)	2.46 (0.77–7.83)
Male sex	86 (40)	0.89 (0.42–1.88)
Hay fever^f^	27 (13)	1.16 (0.40–3.35)
Ever wheeze	52 (26)	0.99 (0.40–2.46)
Medical care and SEP		
Medical insurance coverage	187 (87)	2.44 (0.57–10.49)
Parental education^g^		
High school or less	69 (33)	1.19 (0.51–2.73)
Some college^d^	87 (41)	1.00
College or greater	54 (26)	0.93 (0.35–2.45)
Home characteristics		
Mildew in home	59 (29)	0.59 (0.23–1.51)
Carpet in child’s bedroom	183 (95)	0.76 (0.30–1.91)
Plants in home	72 (34)	1.12 (0.52–2.41)
Pets at home	181 (83)	0.80 (0.32–2.02)
Gas stove in home	168 (79)	1.52 (0.54–4.27)
Current daily smoker in home	29 (13)	0.24 (0.03–1.65)
Family history		
Maternal smoking during pregnancy	24 (13)	0.95 (0.29–3.07)
Parental history of asthma^h^	35 (17)	1.31 (0.51–3.35)

Abbreviations: CI, confidence interval; NA, not applicable.

a Total subject number = 217; subject numbers in the table vary because of missing covariate values.

b Crude estimates with baseline strata for age and sex and community random effects, except model for male sex.

c Could not derive hazard ratio because of small counts in this stratum.

d Reference group, total subject number = 217; subject numbers in the table vary because of missing covariate values.

e Age- and sex-adjusted body mass index.

f Symptoms in the preceding 12 months.

g Categories for parental educational attainment were less than 12th grade or completion of high school, some college or technical school, and completed 4 years of college or some graduate training after college.

h Medical diagnosis of asthma in either biologic parent.

**Table 2:** Community SEP and meteorologic characteristics.

Risk factor^a^	Mean ± SD	Hazard ratio (95% CI)^b^
SEP characteristics		
Percent low education	20.07 ± 5.90	1.07 (0.97–1.18)
Percent male unemployment	4.45 ± 1.05	1.21 (0.71–2.07)
Median household income	39,498 ± 7,003	0.44 (0.17–1.12)
Percent poverty	9.15 ± 3.20	1.11 (0.96–1.29)
Climatic characteristics		
Mean daily average relative humidity (%)		
Fall–winter months	63.08 ± 14.13	1.02 (0.99–1.06)
Summer months	56.89 ± 15.40	1.03 (1.00–1.06)
Annual	59.59 ± 13.76	1.03 (1.00–1.07)
Mean daily maximum relative humidity (%)		
Fall–winter months	78.07 ± 13.46	1.03 (1.00–1.07)
Summer months	75.23 ± 15.67	1.03 (1.00–1.07)
Mean daily average temperature (°C)		
Fall–winter months	13.67 ± 2.15	1.25 (0.82–1.93)
Summer months	18.77 ± 2.39	0.90 (0.72–1.13)
Annual	16.23 ± 1.93	0.96 (0.67–1.38)
Mean daily minimum temperature (°C)		
Fall–winter months	9.12 ± 2.79	1.29 (0.97–1.72)
Summer months	13.35 ± 1.79	1.09 (0.78–1.54)

CI, confidence interval.

a We derived SEP characteristics using data from the 1990 U.S. Census, and meteorologic characteristics using daily data in 1995 from local meteorologic stations in each community.

b All models feature baseline hazard stratified for age and sex, and random effects for community of residence. Estimates are per one unit change in all cases (i.e., % or °C), except for median household income, which is per $10,000.

**Table 3:** Descriptive statistics for exposure to measured NO_2_ pollution (ppb) by community (mean ± SD).

Community	No.^a^	Fall–Winter	Summer	Annual
Alpine	19	17.5 ± 4.1	19.9 ± 5.1	18.7 ± 3.8
Atascadero	12	15.3 ± 8.8	11.0 ± 4.0	13.3 ± 5.7
Lake Elsinore	22	27.3 ± 3.3	17.6 ± 3.1	22.5 ± 3.1
Lancaster	13	21.0 ± 4.1	15.8 ± 3.9	18.4 ± 3.9
Lompoc	27	13.8 ± 4.0	5.4 ± 1.6	9.6 ± 2.5
Long Beach	22	50.0 ± 10.1	34.1 ± 5.6	41.5 ± 6.5
Mira Loma	15	48.4 ± 3.8	37.7 ± 4.2	43.1 ± 2.9
Riverside	26	42.7 ± 7.7	38.3 ± 7.5	40.7 ± 6.3
San Dimas	30	50.6 ± 6.2	51.3 ± 5.7	51.3 ± 4.4
Santa Maria	17	17.4 ± 3.1	11.9 ± 3.6	14.8 ± 2.8
Upland	14	35.5 ± 6.1	46.3 ± 7.1	40.8 ± 6.4

a Number of subjects varied because of invalid measurements during fall–winter and summer months.

**Table 4:** Association between incident asthma and measured NO_2_ pollution with varying levels of control for confounding.

					Model 3: pollution model without relative humidity		Model 4: pollution model with relative humidity	
Exposure	No.^a^	Null model (σ^2^^b^)	Model 1: individual covariates (σ^2^^b^)	Model 2: individual covariates,relative humidity (σ^2^^b^)	HR (95% CI)^c^	σ^2^^b^	HR (95% CI)^c^	σ^2^^b^
Measured NO_2_ pollution								
Fall–Winter	209	0.57161	0.52319	0.16668	1.17 (0.98–1.41)	0.26924	1.29 (1.11–1.49)	0.00016
Summer	204	0.33705	0.26421	0.07770	1.03 (0.86–1.23)	0.25122	1.27 (1.03–1.57)	0.00020
Annual	196	0.33928	0.27278	0.07303	1.10 (0.91–1.33)	0.21705	1.29 (1.07–1.56)	0.00018

Null model contains only community random effects. Model 1 contains Hispanic ethnicity, enrollment group, and medical insurance coverage, with baseline strata for age and sex. Model 2 we further adjusted for community annual mean relative humidity. Model 3 we further adjusted for pollution but did not adjust for community annual mean relative humidity. Model 4 we further adjusted for community annual mean relative humidity.

a Number of subjects varied because of invalid measurements during winter and summer months.

b Values of σ^2^ are the random-effects variance estimates, which represent the residual variation in asthma incidence attributable to the community cluster after we include all other fixed predictors in the model.

c Measured NO_2_ pollution estimates are over a 6.2-ppb exposure contrast, which is the average within-community interquartile range for average annual measured NO_2_.

**Table 5:** Association between incident asthma and measured NO_2_ pollution: within-community versus between-community models.

Measured NO_2_ pollution	No.^a^	HR (95% CI)^b^	σ^2c^	Likelihood ratio test *p*-value^d^
Fall–winter	209	1.29 (1.11–1.49)	0.00016	
Within-community deviation	209	1.32 (0.91–1.92)	0.00016	> 0.1
Community mean		1.28 (1.09–1.51)		
Summer	204	1.27 (1.03–1.57)	0.00020	
Within-community deviation	204	0.99 (0.57–1.72)	0.00019	> 0.1
Community mean		1.32 (1.05–1.66)		
Annual	196	1.29 (1.07–1.56)	0.00018	
Within-community deviation	196	1.31 (0.76–2.26)	0.00019	> 0.1
Community mean		1.28 (1.05–1.57)		

a Number of subjects varied because of invalid measurements during fall–winter and summer months.

b We adjusted models for Hispanic ethnicity, enrollment group, medical insurance coverage, and community annual mean relative humidity, with baseline strata for age and sex. Measured NO_2_ pollution estimates are over a 6.2-ppb exposure contrast, which is the average within-community interquartile range for average annual measured NO_2_.

c Values of σ2 are the random-effects variance estimates, which represent the residual variation in asthma incidence attributable to the community cluster after we included all other fixed predictors in the model.

d Likelihood ratio tests for the null hypothesis that there is no difference between the random-effects models and between/within-community models were not rejected.

**Table 6:** Association between incident asthma and measured NO_2_ pollution with various exclusions to test for confounding by undiagnosed asthma and early chest illness at study baseline.

	All cases (*n* = 217)		Excluding subjects with wheeze at study baseline (*n* = 152)		Excluding subjects with early childhood chest illness (*n* = 180)	
Measured NO_2_ pollution	No.^a^	HR (95% CI)^b^	No.^a^	(95% CI)^b^	No.^a^	(95% CI)^b^
Fall–winter	209	1.29 (1.11–1.49)	146	1.29 (1.06–1.51)	163	1.22 (1.04–1.45)
Summer	204	1.27 (1.03–1.57)	144	1.21 (0.89–1.65)	173	1.26 (1.00–1.58)
Annual	196	1.29 (1.07–1.56)	138	1.28 (1.00–1.64)	170	1.26 (1.02–1.55)

a Number of subjects varied because of invalid measurements during fall–winter and summer months.

b Measured NO_2_ pollution estimates are over a 6.2-ppb exposure contrast, which is the average within-community interquartile range for average annual measured NO_2_.

**Table 7:** Association between incident asthma and mean daily average relative humidity (annual) with and without control for measured NO_2_ pollution.

		HR (95% CI)^a^	
Mean daily average relative humidity (annual)	No.^b^	Model 1: not adjusted for measured NO_2_ pollution	Model 2: adjusted for measured NO_2_ pollution
Fall–winter	209	2.45 (1.11–5.43)	3.75 (1.85–7.62)
Summer	204	1.98 (0.96–4.08)	4.22 (1.58–11.28)
Annual	196	2.09 (1.00–4.35)	3.90 (1.70–8.97)

a We scaled HR values and 95% CIs across the interquartile range in relative humidity across all communities (23.3%). Models contain adjustment for Hispanic ethnicity, enrollment group, and medical insurance coverage, with baseline strata for age and sex. ^b^Number of subjects varied because of invalid measurements during winter and summer months.
